# Transcription factors STAT-4, STAT-6 and CREB regulate Th1/Th2 response in leprosy patients: effect of *M. leprae* antigens

**DOI:** 10.1186/s12879-018-3601-z

**Published:** 2019-01-14

**Authors:** Rajni Upadhyay, Bhavyata Dua, Bhawna Sharma, Mohan Natrajan, Ajai Kumar Jain, Balaji Kithiganahalli Narayanaswamy, Beenu Joshi

**Affiliations:** 1 Department of Immunology, National JALMA Institute for Leprosy and Other Mycobacterial Diseases (ICMR), Tajganj, Agra, 282004 India; 2 Clinical Division, National JALMA Institute for Leprosy & OMD, Tajganj, Agra, 282004 India; 3 Department of Zoology, Jiwaji University, Gwalior, Madhya Pradesh 474001 India; 4 Department of Microbiology and Cell Biology, Indian Institute of Science, Bangaluru, India

**Keywords:** Leprosy, Th1/Th2 cytokine, STAT-4, STAT-6, CREB

## Abstract

**Background:**

Leprosy is an ideal human disease to study T cell regulation as patients show correlation between cytokine skewed Th1-Th2 responses and clinical forms of the disease. The Role of transcription factors on the modulation of Th1 and Th2 responses by *M. leprae* antigens has not been adequately studied. In the present study, we studied the effect of *M. leprae* antigens on transcription factors STAT-4, STAT-6 and CREB and their correlation with Th1/Th2 cell mediated immune responses in leprosy.

**Methods:**

Leprosy patients of both categories of tuberculoid leprosy (BT/TT) and lepromatous leprosy (BL/LL) were selected from the OPD of NJ1L & OMD, (ICMR), Agra and healthy individuals (H) were chosen from the staff and students working in the institute. Peripheral blood mononuclear cells **(**PBMCs) of the study subjects were stimulated with *M. leprae* antigens (WCL, MLSA, and PGL-1). Sandwich ELISA was done in the culture supernatants of healthy and leprosy patients to detect IL-4, IL-10 and IFN-γ. Further, expression of IFN-γ and IL-4 and activation of STAT4, STAT6 and CREB transcription factors in CD4^+^ T cell with or without stimulation of *M. leprae* antigens was investigated by flow cytometry.

**Results:**

Lepromatous leprosy patients showed significantly lower IFN-γ and higher IL-4 levels in culture supernatant and significantly low expression of IFN-γ and higher expression of IL-4 by CD4^+^ T cells than healthy individuals with or without antigenic stimulation. Antigenic stimulation significantly increased IL-10 in BL/LL patients but not in BT/TT patients or healthy individuals. PGL-1 stimulation led to significantly higher activation of STAT-6 in BT/TT and BL/LL patients in comparison to healthy individuals. All the three antigens led to activation of CREB in healthy and BT/TT patients but not in BL/LL patients.

**Conclusion:**

Our findings show that *M. leprae* antigens differentially modulate activation of T cell transcription factors STAT-4/STAT-6 and CREB. These transcription factors are well known to regulate Th1 and Th2 mediated immune response which in turn could play vital role in the clinical manifestations of leprosy. These observations may help to determine how these T cell transcription factors affect the development of immune dysfunction and whether these new pathways have a role in immunomodulation in intracellular diseases like leprosy and TB.

## Background

The prevalence of leprosy at the end of 2017 was 192,713 cases worldwide and the number of new cases reported was 210,671. India still contributes the major percentage of leprosy cases due to continued transmission in the community [1]. The disease shows an interesting immunological phenomenon wherein the host immunity to *Mycobacterium leprae (M. leprae)* dictates the clinical outcome of the disease. Patients with strong cell mediated responses are able to restrict the infection and are grouped into Tuberculoid type (TT) whereas, patients with low cell mediated immunity and high antibody response harbor numerous organisms and are categorized as Lepromatous type (LL). Leprosy has been an extensively studied human bacterial infection in terms of Th1/Th2 immune responses. T helper (Th) cells are classified into Th1 and Th2 cells based on the cytokines secreted by them [2]. Th1 cells predominantly secrete proinflammatory cytokines such as IFN-γ whereas IL-4 and IL-10 cytokines are secreted by Th2 cells. IFN-γ is a crucial cytokine for protection against mycobacterial infections including leprosy***.*** Th1 type of immune response is characteristic of the tuberculoid form of leprosy; conversely, Th2 type immune response is dominant in the lepromatous form of leprosy.

A wide range of well defined transcription factors, including signal transducer and activator of transcriptions (STATs), T-bet, cyclic AMP (cAMP) responsive element binding (CREB) are known to shape the Th1/Th2 differentiation. Lineage commitment to Th1/Th2 is now better understood in terms of transcription factors. Inappropriate induction of Th1/Th2 cell plays an important role in the outcome of the disease. STAT-4 and STAT-6 play important roles in regulating the differentiation of Th cell subsets. STAT-4 is an essential component of the IL-12 signaling pathway and plays an important role in Thl differentiation. Although STAT-4 is expressed both in Th1 and Th2 cells, STAT-4 can only be phosphorylated by IL-12 in Th1 cells as there is marked down-regulation of IL-12Rβ specifically in Th2 cells [3]. However, little is known about the exact mechanism by which STAT-4 activation leads to Th1 differentiation. In contrast to STAT-4, STAT-6 plays a central role in modulating Th2 differentiation. Binding of IL-4 to the IL-4 receptor results in the phosphorylation and dimerization of STAT-6 [4]. Furthermore, CREB, a transcription factor which belongs to the family of basic leucine zipper (bZIP), binds to cAMP responsive element (CRE) and is essential for T cell function and cytokine production [5]. CREB plays various roles in immune function including its role in promoting anti-inflammatory immune responses through inhibition of NF-κB activity, the induction of IL-10, and the generation of regulatory T cells. These anti-inflammatory responses could be protective by inhibiting unwanted inflammation, tissue damage, and autoimmune responses, or they could be pathogenic in the context of infection and tumor immunosurveillance [6].

Role of transcription factors on the modulation of immune responses in leprosy has not been thoroughly studied. Kim et al.*,* have shown IL-12 induced STAT-4 phosphorylation and DNA binding in *M. leprae*-activated T cells in TT but not in LL patients [7]. Regulatory role of CREB in production of IFN-γ is well documented in *M. tuberculosis* infection [8]. Therefore, in the present study we have studied *M. leprae* antigens mediated Th1/Th2 specific T cell transcription factors STAT-4, STAT-6, and CREB activation and cytokine production in leprosy patients and healthy individuals.

## Methods

### Study subjects

Leprosy patients of both the categories of tuberculoid leprosy (TT/BT) (*N* = 15) and lepromatous leprosy (BL/LL) (*N* = 9) were selected from the OPD of National JALMA 1nstitute for Leprosy & OMD (ICMR), Agra (Age range 19-48 yrs). Patients were diagnosed on the basis of clinical and bacteriological criteria and classified according to the immunological scale of Ridley-Jopling [9]. Twelve healthy individuals working in the laboratory were included as healthy controls. All the healthy individuals were clinically free from infections at the time of sample collection and had no history of TB and Leprosy. In addition, they were not contacts of patients, therefore, it is unlikely that they would be harboring the disease**.** Ten milliliter peripheral blood was collected in heparinized vials from all study subjects after taking informed written consent and the study was approved by institutional human ethics committee (Human Ethics Committee meeting of National JALMA 1nstitute for Leprosy & OMD, Agra dated 21.2.2010).

### Antigens

*Mycobacterium leprae* soluble antigen (MLSA), Whole cell lysate (WCL) and Phenolic glycolipid-1 (PGL-1) were procured from the laboratory of Dr. John T Belisle, Deptt of Microbiology, Immunology and Pathology, Colorado State University, (under WHO Contract Number USA NIH-NO1-AI-25469).

### Separation of peripheral blood mononuclear cells (PBMCs) from blood and antigenic stimulation

PBMCs were isolated from buffy coats from healthy donors and leprosy patients (both tuberculoid and lepromatous) by density gradient centrifugation using Ficoll hypaque. Cells were incubated in RPMI-1640 supplemented with 5% heat inactivated FBS (Hyclone, USA), 2 mM L- Glutamine, 100 unit penicillin/ml and 100 μg streptomycin/ml (Sigma, USA) at 37 °C and 5% CO2 in a humidified incubator. Cells were stimulated with MLSA (10 μg /ml), WCL (10 μg /ml) and PGL-1 (15 μg/ml) for 24 h. Antigen doses were standardized earlier in our laboratory for a previous study [10]. In brief, standardization of optimum dose of antigen was done by both MTT and LTT assay using PBMCs of healthy individuals. The optimum dose corresponds to the log phase of the curve generated by these assays.

### Sandwich ELISA

Cytokine estimation was done in the culture supernatant of PBMCs of 15BT/TT, 9BL/LL and 12 healthy donors. Supernatants were collected from PBMC culture after 48 h for IL-4 and IL-10 estimation and after 5 days for IFN-γ estimation. Detection of secreted cytokines was done by commercially available sandwich ELISA kits from R & D systems, Minneapolis, USA.

### Flow cytometric analysis

Effect of *M. leprae* antigens (MLSA, WCL, and PGL-1) on phosphorylated status of STAT-4, STAT-6 and CREB on CD4^+^ T cells and on frequency of IFN-γ, IL-4 producing CD4^+^T was analysed by flow cytometry. In brief 2 × 10^6^ cells/ml were stimulated with standard doses of WCL, MLSA and PGL-1, few cells were kept without stimulation. Plates were incubated for 24 h at 37 °C in 5% CO_2_ with humidified air. Six hours before the termination of incubation, cells were treated with monensin (4 μM, Sigma, USA). After incubation cells were stained with antibodies for surface markers - anti- human CD3 PE Cy5, anti- human CD4 FITC and were incubated for 30 min in dark at 4 °C. Cells were then washed and fixed with 4% formaldehyde in phosphate buffer saline (PBS, pH -7.4). Cells were permeabilized and staining was done for intracellular cytokines and transcription factors. Cells were stained with anti-human IL-4 APC, anti human IFN-γ PE Cy7, anti- human pSTAT-4 PE, anti- human pSTAT-6 Alexa Flour 647 and anti -human pCREB Alexa Flour 647 and incubated for 30 min in dark at 4 °C. All the antibodies for flowcytometry were purchased from BD Biosciences, USA. Stained cells were acquired in BD FACS Aria (BD, San Hose, USA) and the percentage of cells was calculated using FACS Diva Software.

### Statistical analysis

Statistical analysis was done using Prism 3 software (Graph pad version 3, LA Jolla, USA). Data was presented as mean ± SEM. Variation between the groups was calculated by non parametric Mann Whitney test. *P* value less than 0.05 was considered as significant. Values of stimulated PBMCs were normalised by subtracting the unstimulated values for comparison among different study subjects.

## Results

### *M. leprae* mediated secretion of Th1/Th2 cytokines in the culture supernatant of PBMCs of leprosy patients and healthy individuals

PBMCs of leprosy patients (BT/TT, BL/LL) and healthy individuals (H) were stimulated with *M. leprae* antigens (MLSA, WCL and PGL-1) and culture supernatants were collected after 48 h and 5 days. Secreted Th1 cytokine IFN-γ and Th2 cytokines IL-4 and IL-10 were estimated in the supernatant.

**Fig. 1:**
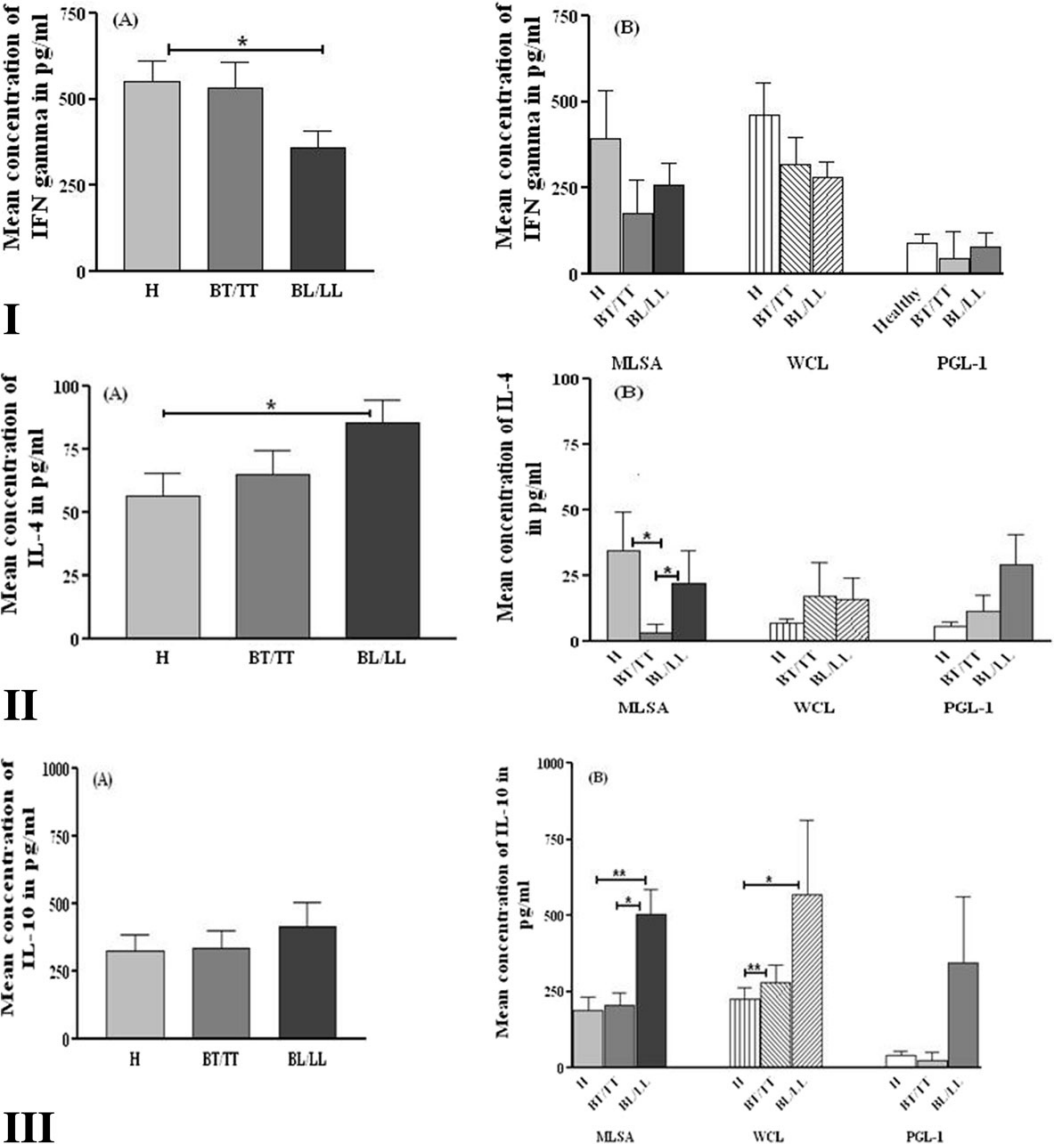
Cytokine levels in culture supernatant of healthy and leprosy patients. Cytokine levels in culture supernatant of unstimulated (A) *M. leprae* antigens stimulated (B) PBMCs of healthy individuals (H), TT/BT and BL/LL patients. PBMCs from the study subjects were cultured with or without antigens for 5 days in RPMI at 37 °C in 5% humidified atmosphere. Supernatants were collected after 48 h and 5 days of culture and cytokine was estimated. Concentration of cytokine was normalized after subtracting values of unstimulated supernatant from stimulated supernatant. **I** = IFNγ, **II** = IL-4, **III** = IL-10. * = *p* < 0.05, ** = *p* < 0.001. MLSA- *M. leprae* soluble antigen, WCL- whole cell lysate, PGL-1- Phenolic glycolipid-1. BT/TT (Borderline tuberculoid/Tuberculoid), BL/LL (Borderline lepromatous/Lepromatous)

In absence of antigens significantly higher production of IFN-γ (*p* = 0.027) was observed in culture supernatant of healthy individuals (H) as compared to BL/LL patients (Fig. 1(I)A). IFN-γ level was higher in response to MLSA, WCL and PGL-1 in culture supernatant of healthy individuals as compared to lepromatous patients and tuberculoid patients. However, no significant difference was noted among any groups in response to all three antigens (Fig. 1(I)B).

Significantly higher basal concentration of IL-4 (*p* = 0.026) was noted in lepromatous patients (Fig. 1(II)A) as compared to healthy individuals. MLSA significantly increased production of IL-4 in lepromatous patients and healthy individuals as compared to tuberculoid patients (*p* value =0.048, 0.0364 respectively). On other hand, PGL-1 stimulation did not show any significant difference in IL-4 levels in healthy individuals and leprosy patients, but the lowest level of IL-4 was observed in healthy individuals (Fig. 1(II)B).

No difference was noted in IL-10 concentration among patients and healthy individuals in unstimulated PBMCs. Significant up regulation of IL-10 was observed post stimulation with MLSA and WCL in culture supernatant of lepromatous patients in comparison to tuberculoid patients (*p* = 0.014 for MLSA, *p* value = 0.009 for WCL) and healthy individuals (*p* = 0.003 for MLSA, *p* = 0.013 for WCL) (Fig. 1(III)A). Decreased secretion of IL-10 was observed after PGL-1 stimulation in healthy individuals and tuberculoid patients but the change was not significant as compared to lepromatous patients (Fig. 1(III)B). No difference in IL-10 production was noted in culture supernatant of healthy individuals and tuberculoid patients after MLSA and PGL-1 stimulation (Fig. 1(III)B).

### *M. leprae* antigens mediated expression of Th1 and Th2 cytokines, STAT and CREB transcription factors in CD4^+^ T cells in leprosy patients and healthy individuals

Interaction of T cell receptor with MHC/antigen complex leads to activation of naïve T cells and its differentiation to Th1 or Th2 cell type expressing IFN-γ and IL-4 cytokines respectively. Role of transcription factor STAT-4 in the induction of IFN-γ by IL-12 has been suggested whereas differentiation of Th2 cells is mediated by STAT6. Transcription factor CREB plays diverse role in immune response. It regulates Th1, Th2 and Th17 type of immune response differentially. Therefore, expression of cytokines IFN-γ, IL-4 and transcription factors STAT-4, STAT-6 and CREB on CD4^+^ T cells with and without stimulation of MLSA, WCL and PGL-1 antigen was studied. PBMCs from leprosy patients and healthy individuals were stimulated with antigens and stained with fluorochrome conjugated anti-CD4 antibody to intracellular cytokines. Cells were acquired by flow cytometer. Analyzed results were presented as mean percentage of cytokines expressing CD4^+^ T cells in blood of healthy individuals and leprosy patients.

#### Expression of IFN-γ and IL-4 in CD4^+^T cells

**Fig. 2:**
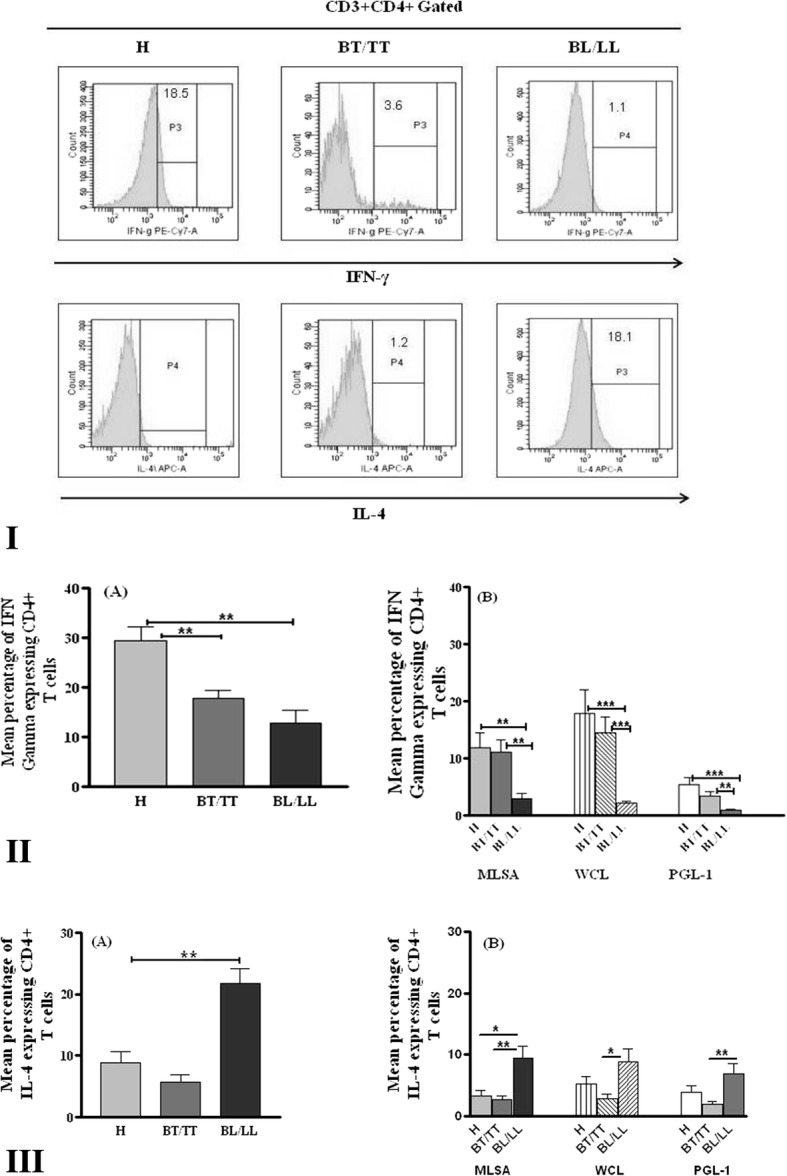
IFN-γ and IL-4 expressing CD4^+^T cells in healthy individuals and leprosy patients. **I** Representative histogram showing CD4^+^T cells expressing IFN-γ and IL-4 in healthy individuals and leprosy patients. **II** Mean Percentage of IFN-γ and **III** IL-4 expressing CD4^+^T cells in healthy individuals (H) and leprosy patients with or without stimulation with *M. leprae* antigens. PBMCs of different study subjects were incubated with medium only (A) or stimulated with MLSA, WCL and PGL-1antigens (B) and incubated for 24 h. After incubation cells were washed and stained with fluorescent labeled anti-CD3, anti-CD4, anti IFN-γ and anti-IL-4 antibodies and acquired in flow cytometer. *p < 0.05 and **p < 0.001 *** = *p* < 0.0001. BT/TT (Borderline tuberculoid/Tuberculoid), BL/LL (Borderline lepromatous/Lepromatous)

Leprosy patients showed significantly reduced IFN-γ expressing CD4^+^ T cells (*p* = 0.005 for BT/TT, *p* = 0.0006 for BL/LL) as compared to healthy individuals at basal level (Fig. 2(II)A). These cells were also significantly higher in healthy individuals than lepromatous patients in response to antigenic stimulation (*p* value = 0.002 for MLSA, *p* = 0.0002 for WCL, *p* < 0.0001 for PGL-1) and tuberculoid leprosy patients also showed significantly higher frequencies of these cells in response to antigenic stimulation (*p* = 0.003 for MLSA, *p* = 0.0001 for WCL, *p* < 0.033 for PGL-1) as compared to lepromatous patients (Fig. 2(II)B).

Basal mean percentage of IL-4 expressing CD4^+^ T cells was significantly higher (*p* = 0.0008) in PBMCs of lepromatous leprosy patients as compared to healthy individuals. Significantly higher IL-4 expressing CD4^+^ T cells were noted in LL patients after **s**timulation with MLSA in comparison to healthy and BT/TT patients whereas significant difference was observed in the expression of these cells in only BL/LL and BT/TT patients after stimulation with WCL and PGL-1(*p* = 0.0057 for MLSA, *p* = 0.0133 for WCL, *p* = 0.0041 for PGL-1) (Fig. 2(III)A & B).

#### Expression of phosphorylated STAT-4 and STAT-6 in CD4^+^T cells

**Fig. 3:**
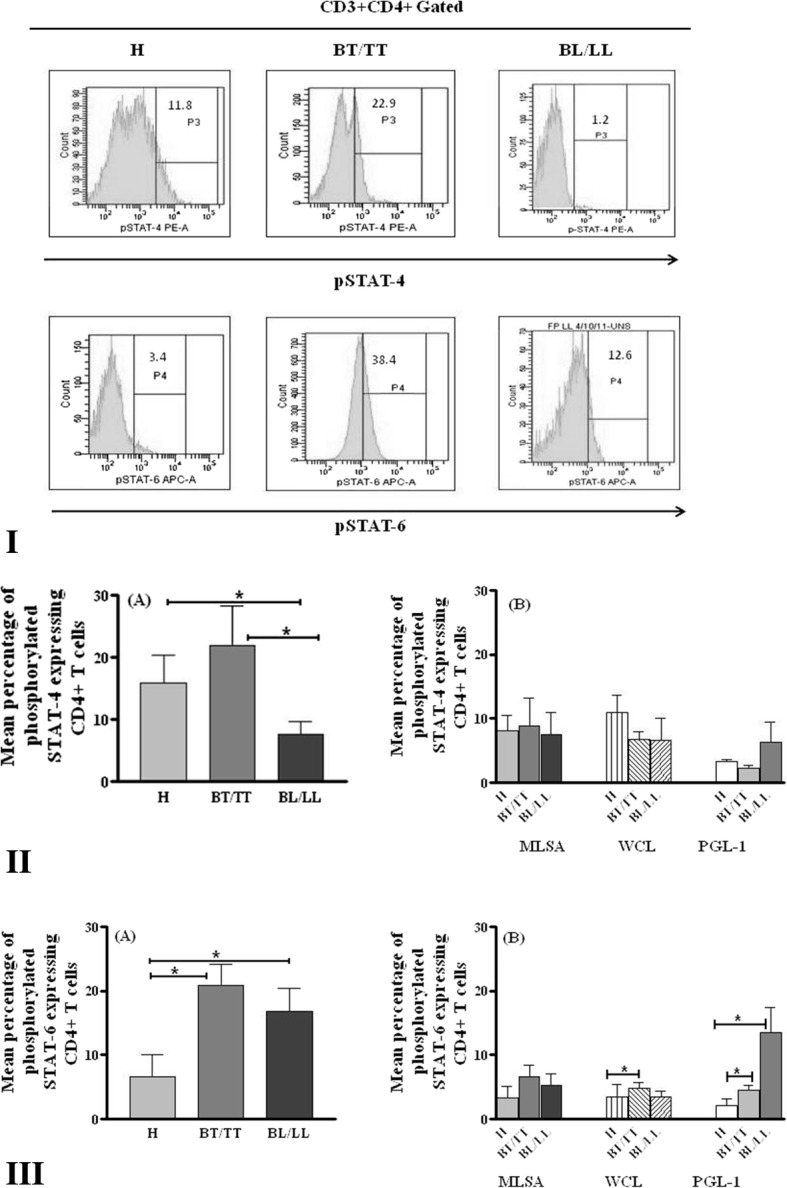
Phosphorylated STAT-4 and STAT-6 expressing CD4^+^T cells in healthy individuals and leprosy patients. **I** Representative histogram showing CD4 + T cells expressing STAT-4 and STAT-6 in healthy individuals and leprosy patients. **II** Mean Percentage of pSTAT-4 and **III** pSTAT-6 expressing CD4^+^T cells in healthy individuals (H) and leprosy patients with or without stimulation with *M. leprae* antigens. PBMCs of different study subjects were incubated with medium only (A) or stimulated with MLSA, WCL and PGL-1antigens (B) and incubated for 24 h. After incubation cells were washed and stained with fluorescent labeled anti-CD3, anti-CD4, anti pSTAT-4 and anti pSTAT-6 antibodies and acquired in flow cytometer. * = *p* < 0.05. BT/TT (Borderline tuberculoid/Tuberculoid), BL/LL (Borderline lepromatous/Lepromatous)

Significantly higher pSTAT-4 expression by CD4^+^ T cells was observed in healthy individuals (*p* = 0.046) and tuberculoid patients (*p* = 0.042) as compared to lepromatous patients when no antigen was added. However, no difference was noted among various groups after antigenic stimulation (Fig. 3(II)A & B).

Mean percentage of pSTAT-6 expressing CD4^+^ T cells was compared among leprosy patients and healthy individuals. Basal pSTAT-6 expression by CD4^+^ T cells was significantly higher in BL/LL and BT/TT patients both in comparison to healthy individuals (*p* = 0.0175, 0.035 respectively) (Fig. 3(III)A). Further, no significant difference in pSTAT-6 expression by CD4^+^ T cells was noted in healthy individuals, BL/LL and BT/TT patients when PBMCs were stimulated with MLSA however, higher pSTAT-6 expression was observed in BT/TT and BL/LL patients than healthy individuals. After WCL stimulation, significantly higher expression of pSTAT-6 by CD4^+^ T cells was noted in BT/TT patients (*p* = 0.0487) as compared to healthy individuals. BL/LL (*p* = 0.007) and BT/TT (*p* = 0.0487) patients showed significantly higher expression of STAT-6 after PGL-1 stimulation when compared to healthy individuals (Fig. 3(III)B).

**Fig. 4:**
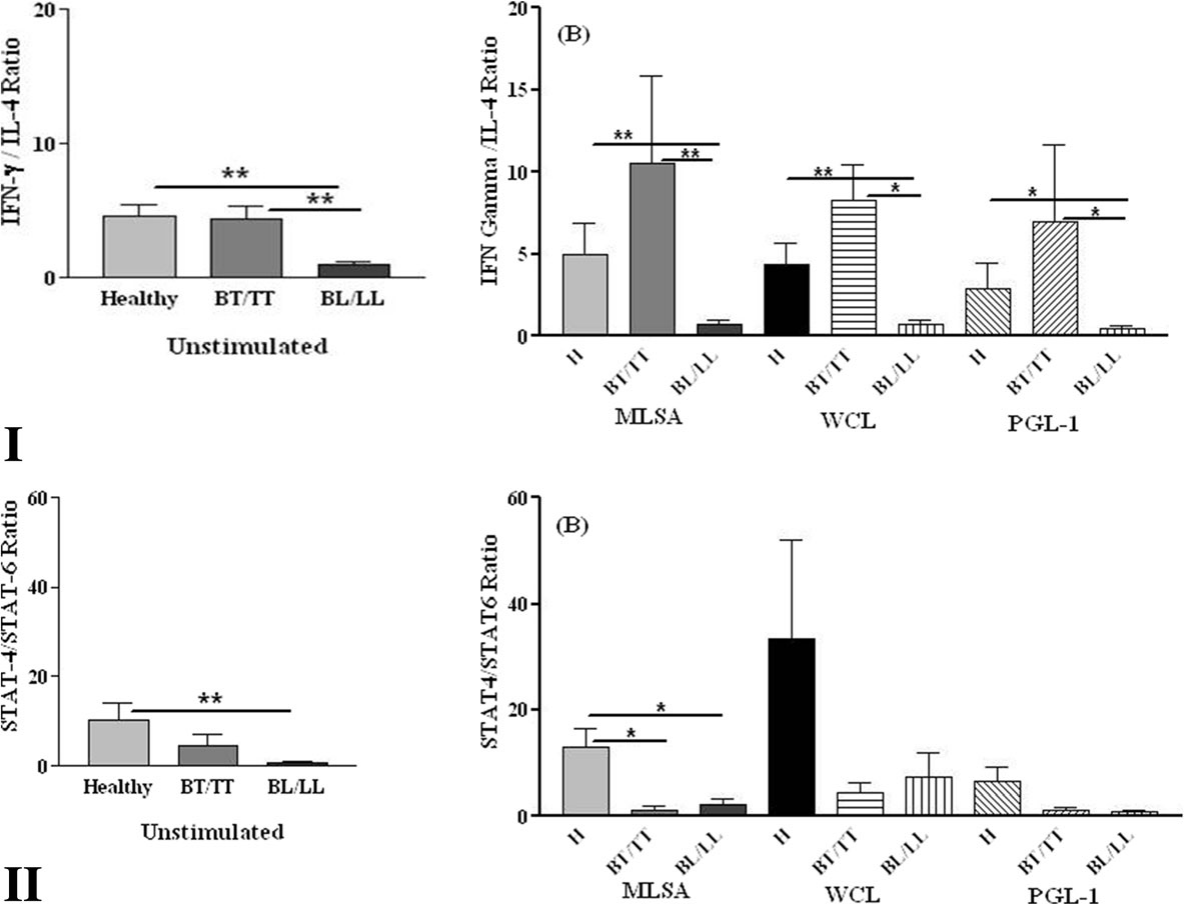
Ratio of IFN-γ/IL-4, pSTAT4/pSTAT6 expressing CD4^+^T cells in healthy individuals and leprosy patients. Ratio of IFN-γ/IL-4 (**I**), pSTAT4/pSTAT6 (**II**) expressing CD4^+^T cells in healthy individuals (H) and leprosy patients with or without stimulation with *M. leprae* antigens. * = *p* < 0.05 and ** = *p* < 0.001. BT/TT (Borderline tuberculoid/Tuberculoid), BL/LL (Borderline lepromatous/Lepromatous)

Ratio of mean percentages of basal and antigen mediated IFN-γ and IL-4 expressing CD4^+^ T cells was significantly higher in healthy and BT/TT patients than BL/LL patients (Fig. 4(I)A & B). However, significantly higher ratio of STAT4/STAT6 expressing CD4^+^ T cells was noted in unstimulated PBMCs in healthy individuals only as compared to BL/LL patients and to both BT/TT and BL/LL patients in response to MLSA (Fig. 4(II)A & B)

#### Activation of CREB in CD4^+^T cells

**Fig. 5:**
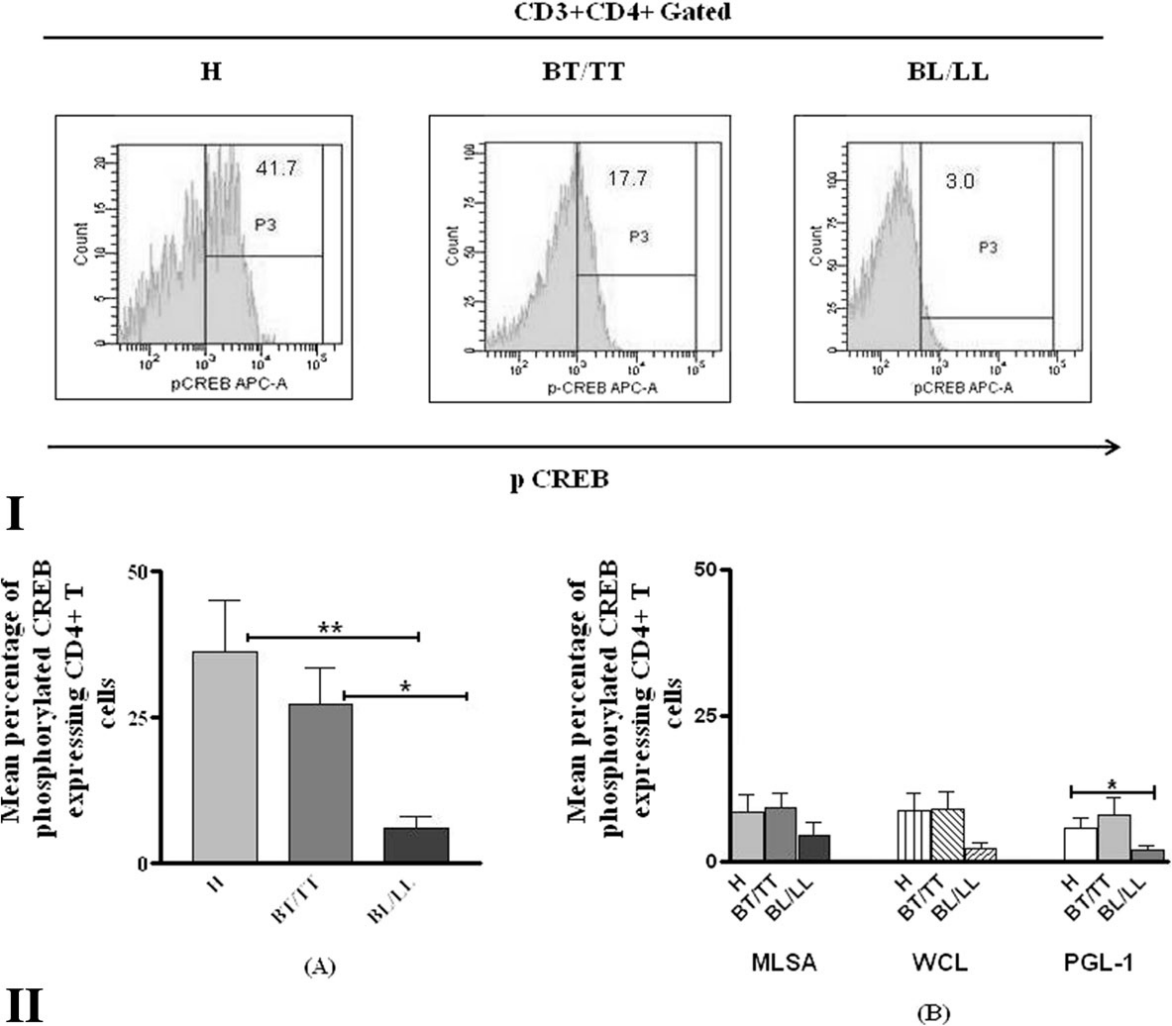
Phosphorylated CREB expressing CD4^+^T cells in healthy individuals and leprosy patients. **I** Representative histogram showing CD4 + T cells expressing CREB in healthy individuals and leprosy patients. **II** Mean Percentage of pCREB expressing CD4^+^T cells in healthy individuals (H) and leprosy patients with or without stimulation with *M. leprae* antigens. PBMCs of different study subjects were incubated with medium only (A) or stimulated with MLSA, WCL and PGL-1antigens (B) and incubated for 24 h. After incubation cells were washed and stained with fluorescent labeled anti-CD3, anti-CD4, anti-pCREB and acquired in flow cytometer. * = *p* < 0.05 and ** = *p* < 0.001. BT/TT (Borderline tuberculoid/Tuberculoid), BL/LL (Borderline lepromatous/Lepromatous)

Expression of activated transcription factor CREB which regulates proliferation and differentiation of T cells was also analyzed after antigenic stimulation. Basal expression of phosphorylated CREB by CD4^+^ T cell was significantly higher (*p* = 0.0039) in PBMCs of healthy individuals and BT/TT patients as compared to BL/LL patients (Fig. 5(II)A). Higher expression of pCREB by CD4^+^ T cells was also noted in healthy individuals and BT/TT patients as compared to lepromatous patients after MLSA, WCL and PGL-1 stimulation. However, significant difference was noted in healthy individuals and BL/LL patients only with PGL-1 (*p* = 0.046) stimulation (Fig. 5(II)B).

## Discussion

In spite of several investigations, the mechanisms of *M. leprae* specific T cell anergy in LL patients are not well understood. This study has been done to correlate differential Th1/Th2 cell mediated immune responses observed in leprosy with known T cell transcription factors (STAT-4, STAT-6 and CREB) ex vivo along with the effect of *M. leprae* antigens (MLSA, WCL and PGL-1) on the modulation of these transcription factors.

Detection of Th1 cytokine IFN-γ and Th2 cytokines IL-4 and IL-10 in the PBMCs confirmed Th1/Th2 polarization of immune response in leprosy patients. Significantly lower basal concentration of IFN-γ was observed in culture supernatant of BL/LL patients than healthy individuals. When IFN-γ production to different antigens was compared, no difference in the cytokine level was noted among all subject categories. This could be due to different cell populations namely, CD4^+^T cells, NK cells and CD8^+^T cells secreting the cytokines in the medium. It was also noticed that very low level of IFN-γ was produced in response to PGL-1 in healthy individuals and in both types of leprosy patients. These observations confirm low immunogenicity of PGL-1. It also shows that IFN-γ might be helping tuberculoid patients to restrict the *M. leprae* growth as IFN-γ was significantly higher in BT/TT than BL/LL patients after stimulation with *M. leprae* antigens. Earlier similar observations were reported in studies done by Misra et al. [11]; Dockrell et al. [12] and Weir et al. [13].

Significantly higher basal level of Th2 cytokines IL-4 was observed in culture supernatant of lepromatous patients than healthy individuals and tuberculoid patients. After MLSA and WCL stimulation, levels of IL-4 were lower in tuberculoid patients than healthy individuals and BL/LL patients which is intriguing. However, it could suggest higher Th1 response in tuberculoid type of patients which may be due to the presence of well developed cell mediated immunity in tuberculoid patients that is directly related with T cell activation and differentiation. Highest production in IL-4 after PGL-1 stimulation is due to Th2 response exhibited by lepromatous patients as well as immunosuppressive nature of PGL-1. These findings show that PGL-1 might be responsible in inducing Th2 response which lowers Th1 response and help in bacterial growth which is seen in LL patients.

Expression of IFN-γ, IL-4 by CD4^+^T cells with and without stimulation of *M. leprae* antigen was also studied by flow cytometry in order to confirm observations noted by ELISA. Similar findings were noted in the case of both IFN-γ and IL-4 secreting CD4^+^T cells. Even more significance is found in flow cytometry data as compared to ELISA results because of its higher sensitivity and T cell specific cytokine secretion. Basal IFN-γ level was significantly higher in healthy individuals than leprosy patients. However, all the three antigens induced increase in IFN-γ expressing CD4^+^T cells in healthy individuals and tuberculoid leprosy patients than in LL patients which is in contrast to findings observed by ELISA. In the culture supernatant several cells like CD4^+^T cells, NK cells and CD8^+^T cells are the source for IFNγ production whereas in flow cytometry we have targeted specific cells, namely CD4^+^T cells hence significant antigen specific response was observed. On the contrary, IL-4 expressing CD4^+^T cells showed significantly higher mean percentage of these cells in BL/LL patients than healthy individuals and BT/TT patients. The study confirms Th2 type of response in the lepromatous pole and intact CMI showing Th1 response (high IFN-γ and low IL-4) in TT/BT to *M. leprae* antigens. All of these results are in concordance with the findings of Misra et al. [11]; Dockrell et al. [12] and Weir et al. [13] which have shown strong Th1 response in tuberculoid patients and Th2 response and T cell unresponsiveness in lepromatous patients to *M. leprae* antigens. Dockrell et al.*,* 1996 showed strong lymphoproliferative response and IFN-γ secretion in response to fractionated cell wall or cytosol and membrane proteins of *M. leprae* in tuberculoid leprosy patients [12]. Our results are also in favor of other two studies done by our group earlier which states suppressive nature of *M. leprae* antigens MLSA, WCL and PGL-1 respectively in Jurkat T cells [10, 14].

As IL-10 can directly inhibit Th1 and Th2 cytokine production at the level of T cell [15, 16], this cytokine was also quantified in culture supernatants**.** All of three antigens induced an increase in IL-10 in BL/LL patients which was significantly higher than healthy individuals and BT/TT patients especially after MLSA and PGL-1 stimulation. Role of IL-10 in induction of phagocytic programme in macrophages but not triggering antimicrobial pathway in leprosy has been earlier reported. This leads to survival of mycobacteria leading to extensive disease in LL [17, 18]. Inhibition of *M. leprae* specific T cell proliferation by IL-10 has been reported [19]. Boussiotis et al.*,* 2000 also reported that increased susceptibility to mycobacterial infection is linked with secretion of IL-10 by T cells [20].

T cell transcription factors regulate specific immune response against pathogen after infection, hence we further studied the activation of different transcription factors STAT-4, STAT-6 after stimulation with *M. leprae* antigens. Role of these factors have not been studied widely in leprosy. Activation of STAT-4 is critical for Th1 differentiation and plays an important role in IL-12 signaling pathway. IL-12 receptor is composed of two β-like chains, IL-12Rβ1 and IL-12Rβ2. IL-12Rβ2 is selectively expressed on Th1 but not Th2 cells. IL-12Rβ2 production and expression is limited to activated T-cells which respond to IL-12 by secreting IFN- γ and is down-regulated by IL-4 and IL-10 [21]. In case of infection with intracellular pathogen development toward the Th1 subset is initiated by stimulation with IL-12 and IFN-γ, which are secreted by dendritic cells and macrophages. This Th1 differentiation is thus linked to activation of the transcription factors STAT-1 and STAT-4 downstream of IFN-γ and IL-12 signaling, respectively. Together with the transcription factors such as nuclear factor of activated T cell (NFAT), adaptor related protein complex 1 (AP-1) and nuclear factor κB (NFκB)] that are activated by TCR engagement, STAT1 induces the expression of the master transcriptional factor of the Th1 subset, T-bet. Subsequently, STAT-4 and T-bet act coordinately to produce large amounts of IFN-γ production in Th1 cells [22]. Higher basal levels of STAT-4 in healthy and BT/TT individuals in our study correlated with significant Th1 response in these individuals and may be responsible for their protective immunity which ultimately prevents the infection in healthy and restricts the same in BT/TT patients. Antigen specific activation of STAT-4 was not noted in our study which suggests that pathway other than STAT-4 could be involved in IFNγ secretion in response to *M. leprae* antigens.

Expression and up-regulation of IL-12Rβ2 in leprosy patients which is correlated with cell mediated immunity has been reported earlier by Kim et al. [7]. They reported IL-12 induced STAT-4 phosphorylation in tuberculoid but not in lepromatous patients after *M. leprae* stimulation. This is due to inability of the lepromatous patients to mount an appropriate Th response to *M. leprae,* which was also noted in our study showing low basal level of STAT4 activation. Watford et al., 2008 has identified one new STAT-4 target Map3K8 that has a rather different function [23]. Map3K8 is an upstream activator of ERK, which is inducible by IL-12 and T cell receptor-dependent signals. Chromatin immunoprecipitation assays have revealed that STAT-4 directly binds the *Map3k8* gene. Deficiency of Map3k8 in T cells interferes with IFN-γ production. Our group had earlier shown the inhibitory effects of *M. leprae* antigens on phosphorylation of MAPKs [10, 24]. Hence it is possible that *M. leprae* activates STAT-4 which leads to phosphorylation of MAPKs which is responsible for cytokine expression thereby killing of *M. leprae*. Deficiency of activation of STAT-4 or MAPKs may lead to low IFN-γ which we have also shown, thereby leading to bacterial survival in LL.

STAT-6 is another important Th2 specific transcription factor. Binding of IL-4 receptor to IL-4 induces the phosphorylation of STAT-6 which further activates GATA-3. GATA-3 in return enhances the expression of Th2 cytokines IL-4, IL-5, IL-10 and IL-13 [25]. STAT-6 is not only implicated in the initiation of Th2 differentiation, but it also contributes to maintenance of the Th2 phenotype [4].GATA-3 also inhibits *Stat4* transcription [26, 27] and directly represses *Ifn-γ* [28]. In our study we noted higher STAT-6 expression in unstimulated PBMCs in patients in comparison to healthy individuals which correlated with higher Th1 response in healthy individuals and suggests role of this transcription factor in the development of disease. Further, significantly higher expression of STAT-6 by CD4^+^T cells in BT/TT patients as compared to healthy individuals after WCL stimulation and in both BL/LL and BT/TT patients as compared to healthy individuals in response to PGL-1 also pinpoints role of STAT-6 in favoring of Th2 response and hence establishing infection. These findings also confirm role of PGL-1 in the immunosuppression seen in leprosy by evoking Th2 response through STAT-6 pathway.

CREB is a transcription factor that regulates diverse cellular responses, including proliferation and differentiation of T cells [29]. Liu et al., 2010 showed that CREB could promote the transcription and production of IFN-γ through binding with the IFN-γ proximal promoter [8]. The proximal IFN-γ promoter contains CRE-like sequences (ACGT) where CREB binds and regulates IFN-γ transcription. Although some studies on Jurkat T cells and transgenic mice suggest that CREB proteins inhibit the transcription of IFN-γ [30, 31], positive regulation by CREB of IFN-γ production by *M. tuberculosis* responsive human T cells has been shown [32]. Reduced amounts of CREB binding to the IFN-γ proximal promoter, and absence or diminished expression of phosphorylated CREB was reported in tuberculosis patients which in turn was responsible for reduced IFN-γ production in TB patients [8, 32]. We also observed similar finding in case of leprosy patients showing significantly higher basal expression of CREB in healthy individuals and BT/TT patients than BL/LL patients. It is inferred from this observation that cross regulatory pathways may be involved in the expression of IFN-γ in leprosy patients as BT patients are able to restrict the *M. leprae* infection. CREB activation may be directly linked to Th 1 cells differentiation. Induction of CREB after *M. leprae* antigens stimulation also supports this fact.

## Conclusion

Our study is an effort to investigate the correlation of T cell polarization observed in leprosy with the expression of transcription factors in a few leprosy patients. This study shows differential expression of T cell transcription factor STAT-4, STAT-6 and CREB in leprosy patients and healthy individuals correlating with Th1 and Th2 cytokine expression. Regulation of cell mediated immune response through transcription factors could play an important role in the clinical manifestation of leprosy. More detailed studies of expression profile of different transcription factor on large number of patients are needed to decipher the in vivo regulatory mechanism of T cell differentiation. These observations may provide new tools to study and monitor patients, to determine how these T cell transcription factors affect the development of immune dysfunction, and to study new pathways to block suppressor mechanisms. The findings may also help in better understanding of defects in cell mediated immunity in leprosy as well as other intracellular infections such as tuberculosis and leishmaniasis.

## Abbreviations

BL: Borderline lepromatous leprosy
BT: Borderline tuberculoid leprosy
CMI: Cell mediated immunity
CREB: Cyclic AMP responsive element binding protein
IL-12R: Interleukin 12 receptor
LL: Lepromatous lepromatous leprosy
LTT: Lymphocyte Transformation Test
MAPK: Mitogen activated protein kinase
MLSA: *Mycobacterium leprae* soluble antigen
MTT: 3-(4,5-Dimethylthiazol-2-yl)-2,5-diphenyltetrazolium bromide
PBMC: Peripheral blood mononuclear cells
PGL: Phenolic glycolipid
pSTAT: Phosphorylated signal transducer and activator of transcription
STAT: Signal transducer and activator of transcription
TCR: T cell receptor
Th: T helper
TT: Tuberculoid tuberculoid leprosy
WCL: Whole cell lysate
